# Molecular subtype specific efficacy of MEK inhibitors in pancreatic cancers

**DOI:** 10.1371/journal.pone.0185687

**Published:** 2017-09-28

**Authors:** Diána Brauswetter, Bianka Gurbi, Attila Varga, Edit Várkondi, Richárd Schwab, Gábor Bánhegyi, Orsolya Fábián, György Kéri, István Vályi-Nagy, István Peták

**Affiliations:** 1 MTA-SE Pathobiochemistry Research Group, Budapest, Hungary; 2 Oncompass Medicine Ltd., Budapest, Hungary; 3 Department of Medical Chemistry, Molecular Biology and Pathobiochemistry, Semmelweis University, Budapest, Hungary; 4 St. István and St. László Hospital, Budapest, Hungary; Cellcuity, UNITED STATES

## Abstract

Pancreatic cancer is an increasing cause of cancer related death worldwide. KRAS is the dominant oncogene in this cancer type and molecular rationale would indicate, that inhibitors of the downstream target MEK could be appropriate targeted agents, but clinical trials have failed so far to achieve statistically significant benefit in unselected patients. We aimed to identify predictive molecular biomarkers that can help to define subgroups where MEK inhibitors might be beneficial alone or in combination. Next-generation sequencing data of 50 genes in three pancreatic cancer cell lines (MiaPaCa2, BxPC3 and Panc1) were analyzed and compared to the molecular profile of 138 clinical pancreatic cancer samples to identify the molecular subtypes of pancreatic cancer these cell lines represent. Luminescent cell viability assay was used to determine the sensitivity of cell lines to kinase inhibitors. Western blot was used to analyze the pathway activity of the examined cell lines. According to our cell viability and pathway activity data on these model cell lines only cells harboring the rare G12C KRAS mutation and low EGFR expression are sensitive to single MEK inhibitor (trametinib) treatment. The common G12D KRAS mutation leads to elevated baseline Akt activity, thus treatment with single MEK inhibitors fails. However, combination of MEK and Akt inhibitors are synergistic in this case. In case of wild-type KRAS and high EGFR expression MEK inhibitor induced Akt phosphorylation leads to trametinib resistance which necessitates for MEK and EGFR or Akt inhibitor combination treatment. In all we provide strong preclinical rational and possible molecular mechanism to revisit MEK inhibitor therapy in pancreatic cancer in both monotherapy and combination, based on molecular profile analysis of pancreatic cancer samples and cell lines. According to our most remarkable finding, a small subgroup of patients with G12C KRAS mutation may still benefit from MEK inhibitor monotherapy.

## Introduction

Despite the recent success of targeted therapies treating several tumor types, pancreatic cancer still has very poor prognosis. According to the data of Globocan 2012, pancreatic cancer is responsible for 331000 deaths per year worldwide and has a mortality: incidence ratio of 0.98 [1]. A projection of cancer deaths in the United States to 2030 ranks this cancer type to the second place, just behind lung cancer [2].

The relatively few types and rarity of alarming symptoms lead to diagnosis at an advanced stage, which makes surgical treatment often impossible, or insufficient [3], thus only a well-chosen systemic therapy could improve the chances of survival.

The genetic landscape of pancreatic cancer is well characterized [4, 5] and dominated by four “mountains of cancer genes”: KRAS (71%), TP53 (49%), CDKN2A (22%) and SMAD4 (20%) [4, 6, 7]. Nonetheless FDA approved only three new treatments in the last 20 years for pancreatic cancer (gemcitabine, erlotinib, nab-paclitaxel), of which the only targeted agent is the EGFR inhibitor erlotinib.

The biggest challenge is the high rate of KRAS mutations, whose direct inhibition -despite all efforts- is still difficult. The use of potent indirect, downstream inhibitors such as MEK inhibitors made no or not significant improvement in overall and progression-free survival, even if the patients with mutant KRAS bearing tumors were analyzed separately [8, 9].

Prahallad and colleagues proved the existence of a feedback loop resulting in the activation of the EGFR/PI3K/Akt pathway when using BRAF inhibitors in colon cancers cell lines [10]. This mechanism was also confirmed in pancreatic cancer cell lines. It was also revealed that MEK inhibitors and PI3K inhibitors have a synergistic effect in certain cases [11, 12]. However the underlying molecular patterns of sensitive and resistant tumors are not clear therefore the prediction of synergetic effect is currently not possible.

The routine molecular profiling of tumors in clinical setting with targeted hotspot next generation sequencing (NGS) panels is more and more common in precision oncology programs of large oncology centers. The results are interpreted by molecular tumor boards to refer patients to targeted clinical trial or indicate target based off-label therapies.

The aim of our research was to analyze if there is a subtype of pancreatic cancer patients based on detailed molecular profile available in clinical settings, which would benefit from MEK inhibitors in monotherapy or in combination with other targeted therapies in clinical trials or off label indications, and to provide scientific rationale to initiate new trials with MEK inhibitors in specific molecular subtypes of pancreatic cancers. We used molecularly profiled pancreatic cell lines as relevant in vitro pharmacological models to examine the activated signaling pathways in the presence of different genetic alterations, than test their different sensitivity to MEK inhibitors alone and in combination with other kinase inhibitor combination therapies. Our main question was whether we could predict the efficacy of mono- or combination therapy in certain subgroups of pancreatic cancer patients based on their kinase mutation/expression pattern and if there is a patient subgroup which yet benefits from MEK inhibitors at all.

## Methods

### Data collection

In our retrospective analysis archived Sanger sequencing and next generation sequencing (NGS) data of one hundred thirty-eight pancreas tumors were collected anonymously from the database of the molecular diagnostic laboratory of Oncompass Medicine Ltd. and used for statistical analysis. In the database, fifty oncogenes and tumor suppressor genes (Ion Ampliseq Cancer HotSpot panel v2) were analyzed in 114 patients by next generation sequencing (IonTorrent platform) and different exons of 13 oncogenes were analyzed in 24 patients by Sanger sequencing. In case of NGS biopsies containing tumor cells over 10% were used and coverage between 1000-fold and 1500-fold was achieved. Variants were analyzed using databases to distinguish somatic and hereditary alterations, no normal tissue or blood samples were used. All the sequencing reactions were carried out between 2012 and 2016. Informed consent was obtained from all patients and the anonymized statistical use of sequence data was approved by the National Medical Research Council which serves as the national ethical review committee in Hungary.

### DNA extraction and next generation sequencing of cancer cell lines

The same method was used for the next generation sequencing of three cell lines as in case of formalin-fixed, paraffin-embedded (FFPE) tumor samples. About 500.000 live, cultured cancer cells were harvested for DNA extraction (QIAamp DNA FFPE Tissue Kit, Qiagen, Cat. No. 56404). DNA library were was prepared using Ion AmpliSeq™ Library Kit 2.0 (Thermo Fisher Scientific, Cat. No. 4475345) according to the manufacturer’s instruction. 207 gene fragments were amplified by multiplex PCR (Ion AmpliSeq Cancer Hotspot Panel v2, Thermo Fisher Scientific, Cat No. 4475346). The target sequences were partially digested followed by barcode adapter ligation using Ion Xpress™ Barcode Adapters. Then the libraries were purified using Agencourt^TM^ AMPure^TM^ XP Reagent for the next step of clonal amplification. The pure DNA library was eluted with DNase free water, which does not disturb any downstream methods. Purified DNA library were quantified using Agilent Bioanalyzer DNA chip (High Sensitivity DNA Kit, Kromat Kft, Cat No. 5067–4626) and sequenced on Ion 318 Chip by Ion PGM equipment in the laboratory of Seqomics Kft (Mórahalom, Hungary). The average depth coverage of the amplicons was about 200 000 reads per sample. Variants detected in the libraries were listed in VCF (Variant Call Format) files then annotated and analyzed by the company’s own software. Briefly, union of two VCF files are used as the input of the software, as two parallel NGS takes place from the same tumor sample. These files contain raw data from the sequencing: the detected variants on genomic level, the corresponding quality values, details about the used settings and previous filtering methods. The software annotates the exonic variants on coding DNA and protein level and calculates an artificial confidence score based on selected important quality measures. The final mutation list was evaluated by considering quality scores and filtering out false positive variants.

### Cell culturing

Pancreas adenocarcinoma cell lines MiaPaCa2, BxPC3 and Panc1 obtained from ATCC were cultured in DMEM supplemented with 10% (V/V) fetal bovine serum (FBS, Lonza), 2.5% horse serum (HS, Lonza) and 1% antibiotic mix (MZ, MycoZap Plus-CL, Lonza), RPMI supplemented with 10% FBS and 1% MZ and DMEM supplemented with 10% FBS and 1% MZ respectively in humidified atmosphere of 37°C and 5% CO_2_.

### Kinase inhibitors

Trametinib, afatinib, erlotinib were purchased from Selleckchem (Selleckchem, Munich, Germany), PD0325901, refametinib (RDEA119), triciribine (MK2206) and selumetinib (AZD6244) were purchased from ChemieTek (Indianapolis, IN, USA).

### Cell viability assay and drug synergism

Cell viability was measured with the CellTiter-Glo Luminescent Cell Viability assay (Promega). 1000 per well of the cultured cell lines were seeded into white 96-well plates. Cell lines were left overnight to attach, then treated with decreasing concentrations of trametinib, afatinib, triciribine and the combination of trametinib+afatinib and trametinib+triciribine in duplicates. The final DMSO concentration was 0.2% or less. 72 hours after treatment, appropriate amount of CellTiter-Glo Reagent was added to the cell culture medium in each well. After about 2 minutes shaking, plates were incubated in dark at room temperature for 10 minutes. Luminescent signal was recorded by BioTek Synergy 2 Multi-Mode Microplate Reader. Each experiment was repeated at least three times.

Potential drug synergism was confirmed and combination index at different effective doses (ED) was calculated with Compusyn software, which is based on the Median-Effect Principle and the Combination Index-Isobologram Theorem [13]. Combination indexes generated by Compusyn indicate drug synergism under 1 and additive effect between 0.75 and 1.25 [14]. In this research combination indexes were calculated in a constant concentration ratio of the drugs used.

### Western blot analysis

Cells were grown to 90% confluence in 6 well plates and were treated with 10 nM trametinib in complete medium. After treatment, cells were washed with ice-cold PBS and lysed in lysis buffer (50 mM Tris (pH 7.4), 150 mM NaCl, 1% (V/V) NP-40, 2 mM EDTA, 2 mM EGTA, 50 mM NaF, 1 mM dithiothreitol, 1 mM sodium-ortovanadate and protease inhibitor cocktail (Calbiochem) for 30 minutes on ice. Lysates were centrifuged with 13 000 g at 4°C for 15 minutes and supernant was used for analysis. 10 μg protein samples were subjected to SDS-PAGE and electrotransferred to polyvinylidene-difluoride (PVDF) membranes. Membranes were incubated with the diluted primary antibodies [(total EGFR (clone D38B1, Cat. No. 4267, dilution 1:4000), phospho-Tyr1068 EGFR (clone D7A5, Cat. No. 3777, dilution 1:1000), total Akt (clone 40D4, Cat. No. 2920, dilution 1:4000), phospho-Ser473 Akt (clone D9E, Cat. No. 4060, dilution 1:2000), total ERK 1/2 (clone 3A7, Cat. No. 9107, dilution 1:2000), phospho-Thr202/Tyr204 ERK 1/2 (clone D13.14.4E, Cat. No. 4370, dilution 1:8000) monoclonal antibodies were purchased from Cell Signaling Technology (Danvers, MA, USA) and α-tubulin (clone DM1A, Cat. No. T9026, dilution 1:40000) monoclonal antibody was purchased from Sigma-Aldrich (St. Louis, MO, USA)] at 4°C overnight, and with horse radish peroxidase (HRP) conjugated secondary antibodies for 1 h at room temperature. Bands were visualized by Enhanced Chemiluminescence (ECL) detection system (Perkin Elmer, Waltham, MA, USA) and quantified by ImageJ v1.48 software. Every experiment was carried out at least 3 times.

## Results

### Based on next generation sequencing of 50 genes the molecular profile of MiaPaCa2, BxPC3 and Panc1 cell lines together represent more than the third of pancreatic cancer types

We found the mutation of KRAS in 76.8% of the cases. In 68.9% of the examined tumors, the mutation affected the hotspot at codon 12. Other alterations were found in codon 19 (0.7%), codon 13 (0.7%) and codon 61 (6.5%) the remaining about 23% of the patients had tumors with wild type KRAS. KRAS G12D mutation represented the highest percentage (31.2%) and G12C change was relatively rare (1.5%). The rest of codon 12 mutation divided between G12V (21.7%) and G12R (14.5%).

**Fig 1:**
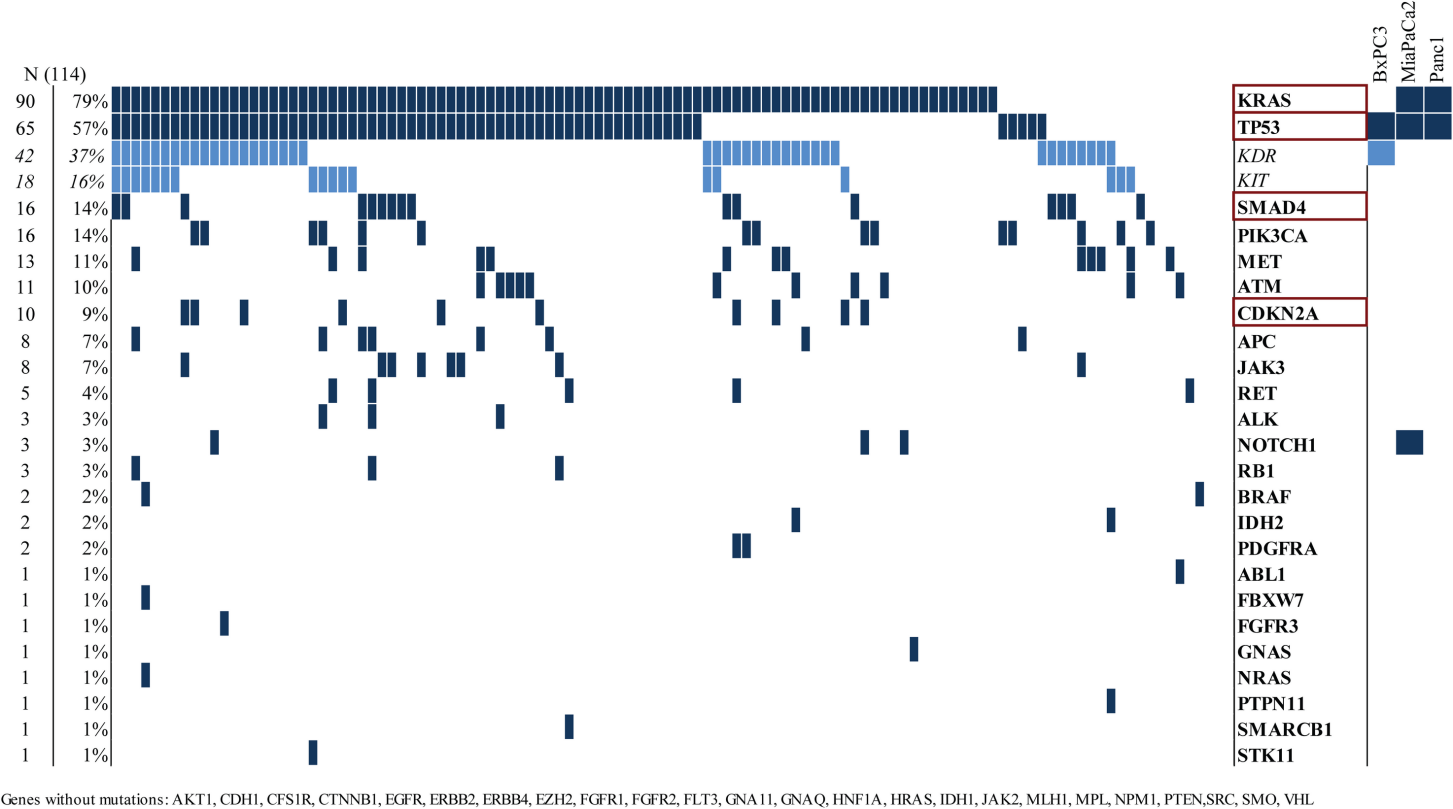
Next-generation sequencing analysis of pancreatic tumors. The mutations (nonsense, missense and frameshift) found in coding region of the 50 examined genes (rows) in pancreatic cancers. Samples (columns, N = 114) are arranged to emphasize coexistence of mutations. Germline mutations found in KDR and KIT genes are marked with different color. Cell lines are indicated, to show the covered percentage of population.

Mutations (missense, nonsense and frameshift) found with NGS of the examined genes and their combined presence is shown in (Fig 1). Based on the next generation sequencing results of the 3 cell lines the following mutations were found: MiaPaCa2 (homozygote mutations in KRAS G12C, TP53 R248W, NOTCH1 L2457V), Panc1 (homozygote mutation in TP53 R273H and heterozygote mutation in KRAS G12D) and BxPC3 (homozygote mutation in TP53 Y220C and KDR Q472H). According to the four most relevant genes (based on COSMIC database) KRAS, TP53, CDKN2A and SMAD4, our in vitro tumor model covers the mutational status of 51/114 (44.4%) pancreas tumor biopsies. If we only take KRAS mutations into consideration MiaPaCa2 (1.5%), Panc1 (31.2%) and BxPC3 (23%) represent more than the half of investigated pancreatic cancers (Fig 1).

It should be noted, that like BxPC3, 42 of the sequenced 114 tumors harbored Q472H mutation in KDR gene. Q472H is frequently observed in melanomas as a germline mutation being associated with increased KDR phosphorylation. While the mutation of NOTCH1 [15] in MiaPaCa2 is not lying within any functional domain and has no known effect on its activity, we didn’t deal with it. CDKN2A and SMAD4 mutations were found in less cases than indicated in COSMIC database, but this can be due to the method, because only the most relevant amplicons of these tumor suppressor genes were sequenced. The full data of the examined amplicons can be found on the manufacturer’s homepage (https://www.thermofisher.com/order/catalog/product/4475346).

### Single MEK inhibitor treatments are very effective in pancreatic cancers harboring the rare G12C mutation in KRAS, while combination therapies can be effective in other subtypes

**Fig 2:**
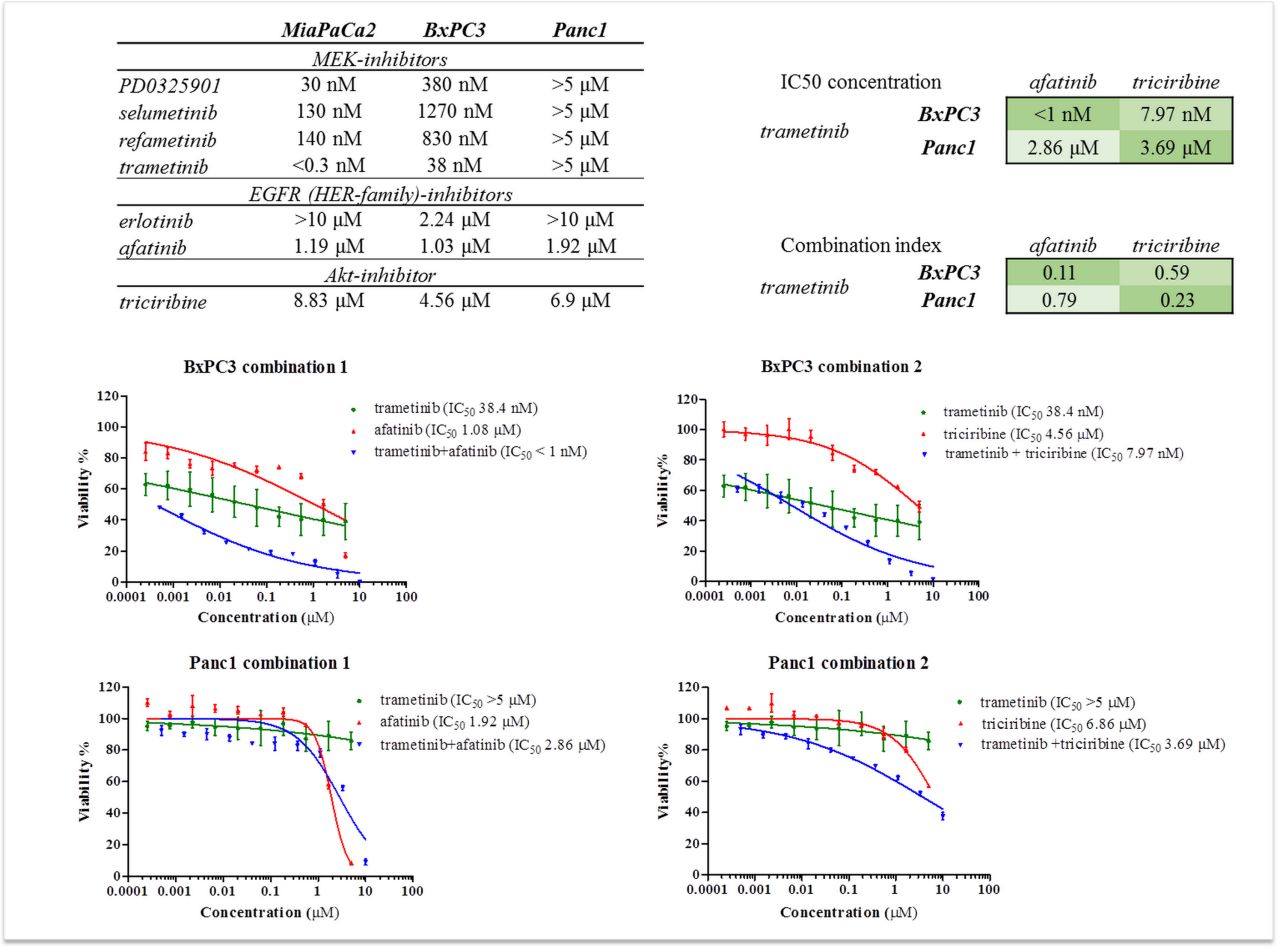
Results of viability assays on pancreatic cancer cell lines. (A) IC_50_ concentrations of MEK, EGFR and Akt inhibitors measured on MiaPaCa2, BxPC3 and Panc1 cell lines (B) IC_50_ curves of MEK inhibitor (trametinib), Akt inhibitor (triciribine) and EGFR inhibitor (afatinib) treatment and MEK+Akt (1:1) -/MEK+EGFR (1:1) inhibitor combination therapy on BxPC3 and Panc1 cell lines, curves were generated with GraphPad Prism version 7.00 for Mac (La Jolla, CA, USA) software (C) IC_50_ concentration of different drug combinations applied in constant ratio (1:1) on BxPC3 and Panc1 cell lines and combination indexes of the same drug combinations calculated with Calcusyn.

We analyzed the inhibitory effect of four frequently used MEK inhibitors. In our in vitro model MiaPaCa2 (KRAS-G12C) showed a very high sensitivity to these inhibitors (Fig 2) The KRAS wild type BxPC3 cell line showed moderate sensitivity while in case of Panc1 (KRAS-G12D), all the MEK inhibitors proved to be absolutely ineffective. The lowest IC_50_ concentration was experienced when we treated MiaPaCa2 and BxPC3 cells with the drug trametinib (Fig 2A). In order to increase efficiency, we first combined trametinib with an EGFR inhibitor. Afatinib was much more effective in vitro than erlotinib, which is the FDA approved drug in pancreatic cancer therapy (Fig 2A), therefore we used afatinib in combination with trametinib. We could reach extreme low clinically relevant IC_50_ values and strong synergic effect (CI: 0.11) in the KRAS wild type BxPC3 cell line. In case of Panc1 we observed only additive effect and the IC_50_ concentrations were very high even in combinations. We also measured the effect of trametinib in combination with the Akt inhibitor triciribine. (Fig 2B and 2C) This combined application of the two drugs reduced the total applied dose in case of both cell lines (BxPC3 and Panc1), and were synergistic in both cell lines particularly in Panc1. But the absolute IC_50_ concentration of trametinib, was not under the clinically applicable limit in this cell line.

### The activated signaling pathway and the inhibitory effect of trametinib depends on the mutation present in KRAS and the expression level of EGFR protein

Western blot analysis was used to examine the expression and phosphorylation of EGFR and downstream ERK and Akt proteins in the three cell lines.

The MiaPaCa2 cell line showed extremely low level of EGFR expression and activation. Furthermore the G12C mutant KRAS activated primarily the MEK/ERK pathway and the feedback activation of the EGFR-Akt pathway was expected to be less pronounced.

BxPC3 cells exhibited the highest EGFR expression and activity. While–probably due to the wild type KRAS- ERK and Akt phosphorylation were in balance but the significant EGFR activity could lead to the activation of the EGFR-Akt feedback loop upon MEK inhibition–as assumed by the previously observed synergism of EGFR or Akt and MEK inhibitors in this cell line.

**Fig 3:**
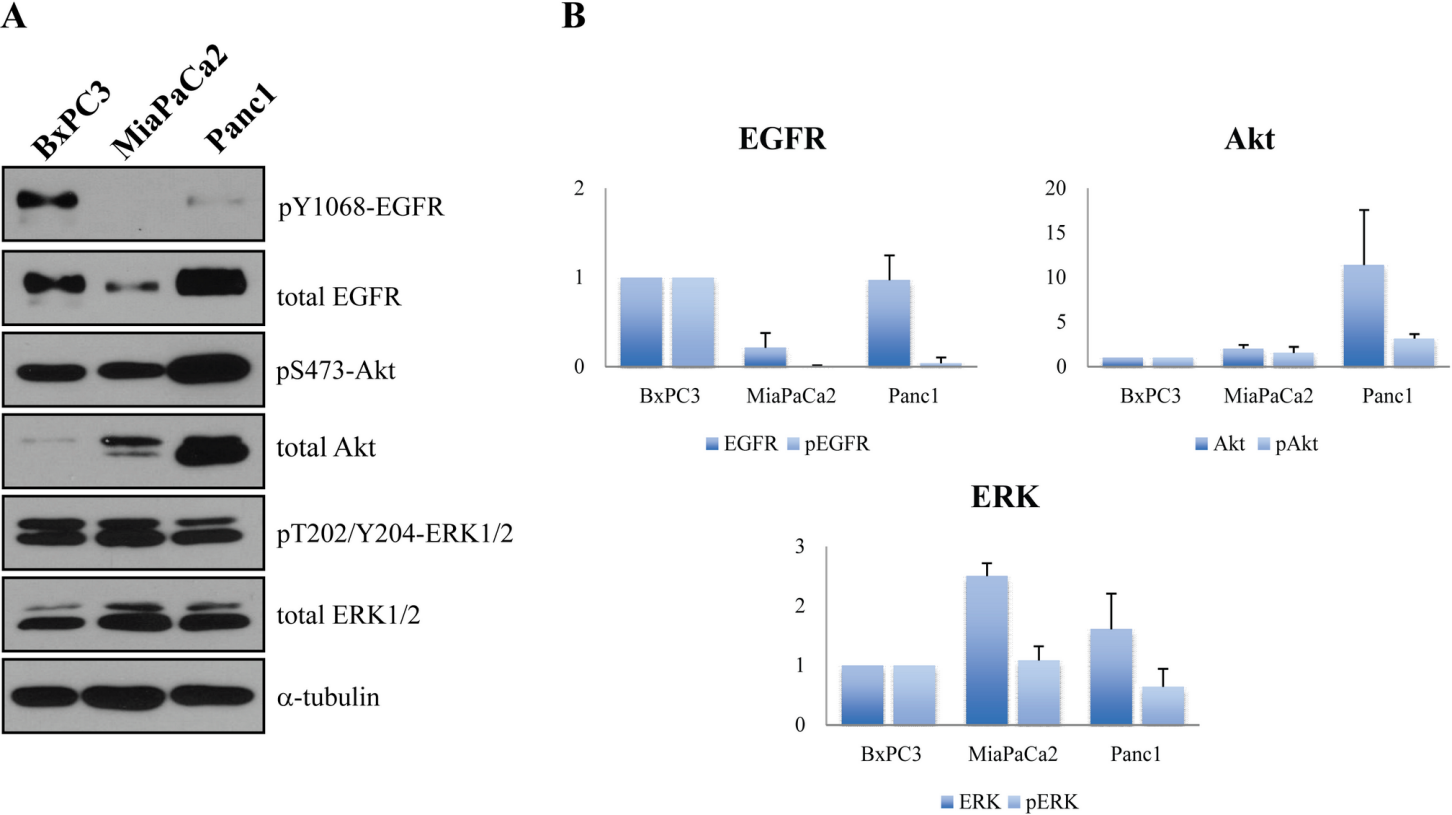
Protein expression and phosphorylation analysis of the used pancreatic cancer cell lines. (A) EGFR and pY1068 EGRF, Akt and pS473 Akt, ERK1/2 and pT202/Y204 ERK were analyzed with SDS page/Western blot method. (B) The expression and phosphorylation of all proteins were compared to the KRAS wild type cell line, BxPC3. α-tubulin was used as loading control. (Original Western blot images: S1A and S1B Fig).

Whereas Panc1 cells expressed high amount of EGFR, its activity wasn’t prominent. However, both expression and phosphorylation of Akt were remarkable in the KRAS G12D mutant Panc1, in turn the activity of ERK was the lowest of all cell lines (Fig 3). This indicates that G12D mutation in KRAS protein activates mainly the PI3K/Akt pathway rather than MEK/ERK signaling, which is in line with the effective growth inhibition of the MEK+Akt inhibitor combination in this cell line (Fig 2).

**Fig 4:**
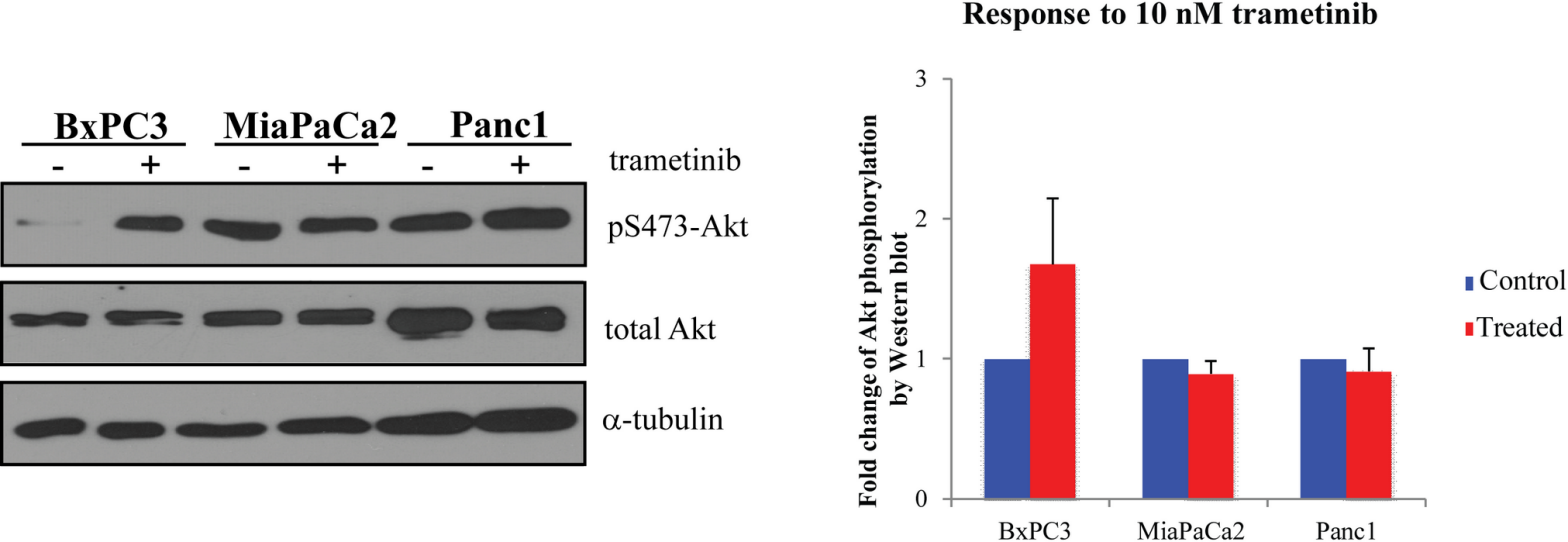
Response of pancreatic cancer cell lines to trametinib treatment. Total Akt level and Akt activation status (pS473) were analyzed by Western blot. A representative blot and graphic evaluation of 3 independent experiments. α-tubulin was used as loading control. (Original Western blot image: S1C Fig).

Next we analyzed the effect of trametinib on EGFR and Akt activity to corroborate the presence of a potential EGFR-feedback activation loop. In line with our hypothesis, trametinib treated MiaPaCa2 cell line showed no increase in Akt activity when compared to the control sample. However, the EGFR overexpressing BxPC3 cell line responded with an increased Akt activity to trametinib treatment. While Panc1 showed remarkable baseline Akt activity both in control cells and trametinib treated samples, further increase due to the treatment wasn’t observed. These observations are in concert with only MiaPaCa2 being highly sensitive to single MEK inhibitor treatment (Fig 4).

## Discussion

In our database of NGS profiles of 114 pancreatic cancer patients, the molecular subtype distribution was similar to COSMIC (cancer.sanger.ac.uk) database [16]. KRAS and TP53 mutation occurred frequently together, while CDKN2A and SMAD4 not. Only one of the tumors harbored mutations in all of the four genes. However 37% of tumors contained a (Q472H) mutation in the KDR gene that was previously observed to be a germline mutation in melanomas, non-small cell lung cancers and Ewing sarcomas in association with KDR phosphorylation or microvessel density [17–19]. This mutation was also found in the BxPC3 cell line, but the analysis of its significance was not the objective of our work.

The examined cell lines represented the half of KRAS mutated pancreatic cancer cell types. According to our NGS analysis, the most prevalent mutation in pancreatic cancers is G12D. G12C is much less frequent in the Caucasian patients and may be associated with different biological characteristics as it was found to be associated with the worst prognosis in non-small cell lung cancer (NSCLC) patients [20]. Interestingly, in Japanese population, G12C mutation occurs more frequently: it was found in the 63% of pancreatic tumors [21].

Although in Caucasian population G12C mutation in pancreatic cancers seems to be less important, their unfavorable prognosis and their different incidence in Japanese population make it a major target in this type of cancer, too.

Besides, based on COSMIC database, G12C is the most frequent mutation at codon 12 in lung cancers (39%) and also often occur in colon cancers (10,3%) [16].

In our in vitro pharmacological model, four MEK inhibitors, two EGFR inhibitors and one Akt inhibitor were tested. We found that MEK inhibitors, particularly trametinib were the most effective on MiaPaCa2 cell line. In this case, we could achieve significant growth inhibition with single MEK inhibitor treatment. This finding is consistent with the data of Pettazzoni et al., where they found no synergism with the combination of selumetinib and erlotinib [11], but their research did not focus on the molecular background of this phenomenon.

Erlotinib is the only targeted agent with FDA approval in pancreatic adenocarcinomas, but Walters and colleagues found HER-2 to be also an important overexpressed protein in pancreatic cancers [22]. Afatinib is a pan-HER inhibitor and showed a greater inhibitory effect in vitro, hence we used it in combination with the MEK inhibitor trametinib. Strong synergistic effect was only revealed on the KRAS wild type BxPC3, while the combination of an Akt inhibitor (triciribine) with trametinib resulted in synergistic inhibitory effect on both BxPC3 and Panc1 cell lines.

In an isogenic colon cancer model, Modest et al. found different ERK activation associated with certain KRAS subtypes [23]. The different pathway activation by KRAS subtypes has been published by Ihle et al [24]. They found mainly MEK inhibitor sensitivity in case of KRAS G12C NSCLC cell lines, while G12D mutant cell lines were rather sensitive to PI3K inhibition. Prahallad et al. proved the feedback activation of EGFR when using BRAF inhibitors [10], which was also observed in pancreatic cancers with MEK inhibitors [12]. It was also revealed that this loop had a major role in cancer types with high EGFR expression [10]. However we observed the best effect of MEK inhibitors (an extremely low IC_50_ concentration in case of trametinib) on MiaPaCa2 cell line, which (apart from the lowest EGFR activation) had KRAS-G12C mutation. Bloomston et al observed EGFR overexpression in 69% of pancreatic tumor tissues[25], which indicates that one third of pancreatic cancers may have lower EGFR expression like MiaPaCa2.

The lower sensitivity of BxPC3 and Panc1 cells to MEK inhibition can be also explained by their different EGFR protein expression and activation. The G12D mutant Panc1 cell line had greater constitutive Akt activity, which could be caused directly by the constitutive activation of the G12D mutant KRAS, so in this case, the combination with an EGFR inhibitor was ineffective. The BxPC3 cell line with wild type KRAS had equally active MEK/ERK and PI3K/Akt pathways, potentially dependent on the high EGFR activity. In this case, the feedback loop can also have potentially a greater importance. In turn, both combinations (MEK+EGFR inhibition and MEK+Akt inhibition) raised synergistic effect in case of BxPC3. This hypothesis was also confirmed by the analysis of Akt activity under trametinib effect.

**Fig 5:**
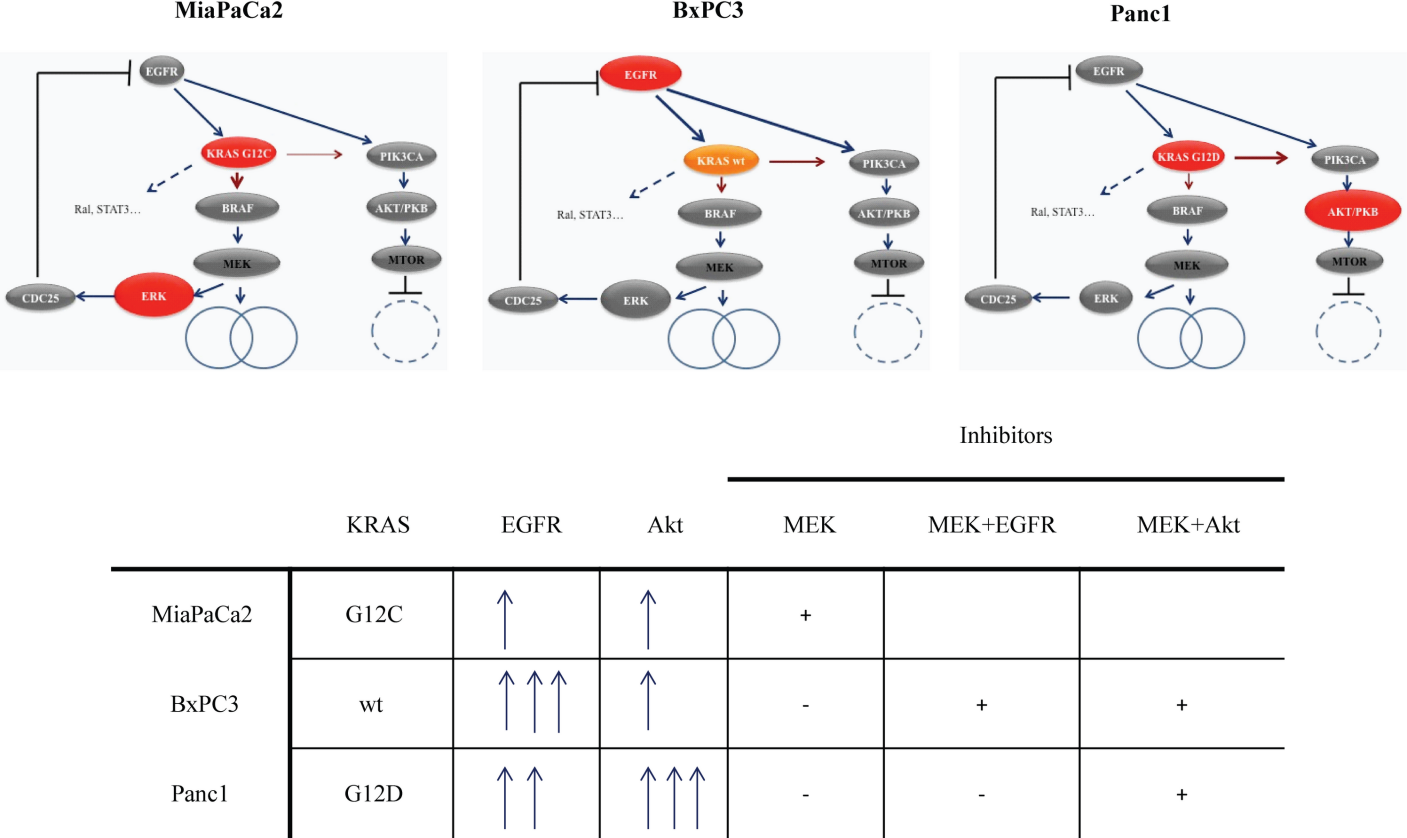
The in vitro pancreatic cancer cell line model. This model is based on our protein expression and phosphorylation measurements and viability assays. MiaPaCa2 cell line with KRAS G12C mutation and low EGFR level is highly sensitive to trametinib treatment, combination with other drugs is not necessary and only increases drug toxicity. In case of BxPC3 cell line with wild type KRAS and high EGFR level/phosphorylation the feedback activation of EGFR/PI3K/Akt to trametinib treatment has a great impact, therefore combination of the MEK inhibitor with EGFR or Akt inhibitor both results drug synergism. Panc1 shows resistance to MEK inhibitors and the combination with the EGFR inhibitor afatinib does not decrease its IC_50_ concentration to an appropriate level. Our model shows, that the presence of G12D mutation (which activates PI3K/Akt pathway) and the high expression of Akt protein both indicate the use of MEK+Akt inhibitor combination.

The synergetic combination of MEK and PI3K/Akt or EGFR inhibitors (erlotinib and lapatinib) has been published before [11, 26, 27] and many ongoing clinical trials can be found with these combination therapies. Although our study represents only three cell lines, the important effect of KRAS G12D or G12C mutations and protein expression on sensitivity to MEK inhibitors and their combinations were never observed before in case of pancreatic cancers (Fig 5).

Our work highlights that like NSCLCs, KRAS mutant pancreatic adenocarcinomas cannot be regarded as a homogeneous group. Cell lines with G12C mutations may be more sensitive to single MEK inhibitor treatment in multiple tumor types. As the intensified side effects observed when using combination therapies [28] can limit their use, it is very important to find a population, where monotherapy can be feasible. KRAS G12C mutation leads to the worst prognosis observed in NSCLCs, too [20], so targeted therapies may have an emerging role in these tumors. A study also revealed the better effect of selumetinib in case of G12C and G12V mutant lung cancers [29]. KRAS G12C inhibitors also offer a very promising therapeutic opportunity, but currently their use is only in research phase [30].

Moreover, it is also necessary to find the most beneficial combination therapy for other molecular subtypes. Based on our result, combination of low concentrations of EGFR inhibitors and MEK inhibitors may be clinically relevant in EGFR expressing KRAS wild type pancreatic cancers, which may represent 23% of pancreatic cancers, in addition to the 2% of G12C KRAS mutants who may respond to MEK inhibition alone. In the case of G12D KRAS mutant cancers, Akt inhibitors may sensitize to the MEK inhibitors but it seems we will need better compounds to be successful in the clinical setting.

It also has to be noted that patients with different KRAS status and protein expression can have a benefit from different combination therapies. G12R and G12V mutations are also important and frequent, but their role was not investigated in this study. Further, the significance of other parallel driver alterations in other molecular subtypes—which were confirmed wild type in the investigated subtypes–will have to be investigated. Based on our results, we propose the combination of NGS sequence data with EGFR expression analysis in order to find the most beneficial treatment in pancreatic cancers.

## Supporting information

S1 Fig Original Western blot images. Figure A, B and C represent the original Western blot images used in Fig 3. and Fig 4.
(TIF)
