# Supplementary figures and images for: Molecular subtype specific efficacy of MEK inhibitors in pancreatic cancers
Source: PLoS One. 2017 Sep 28;12(9):e0185687. doi: 10.1371/journal.pone.0185687 (PMC5619833; doi:10.1371/journal.pone.0185687)

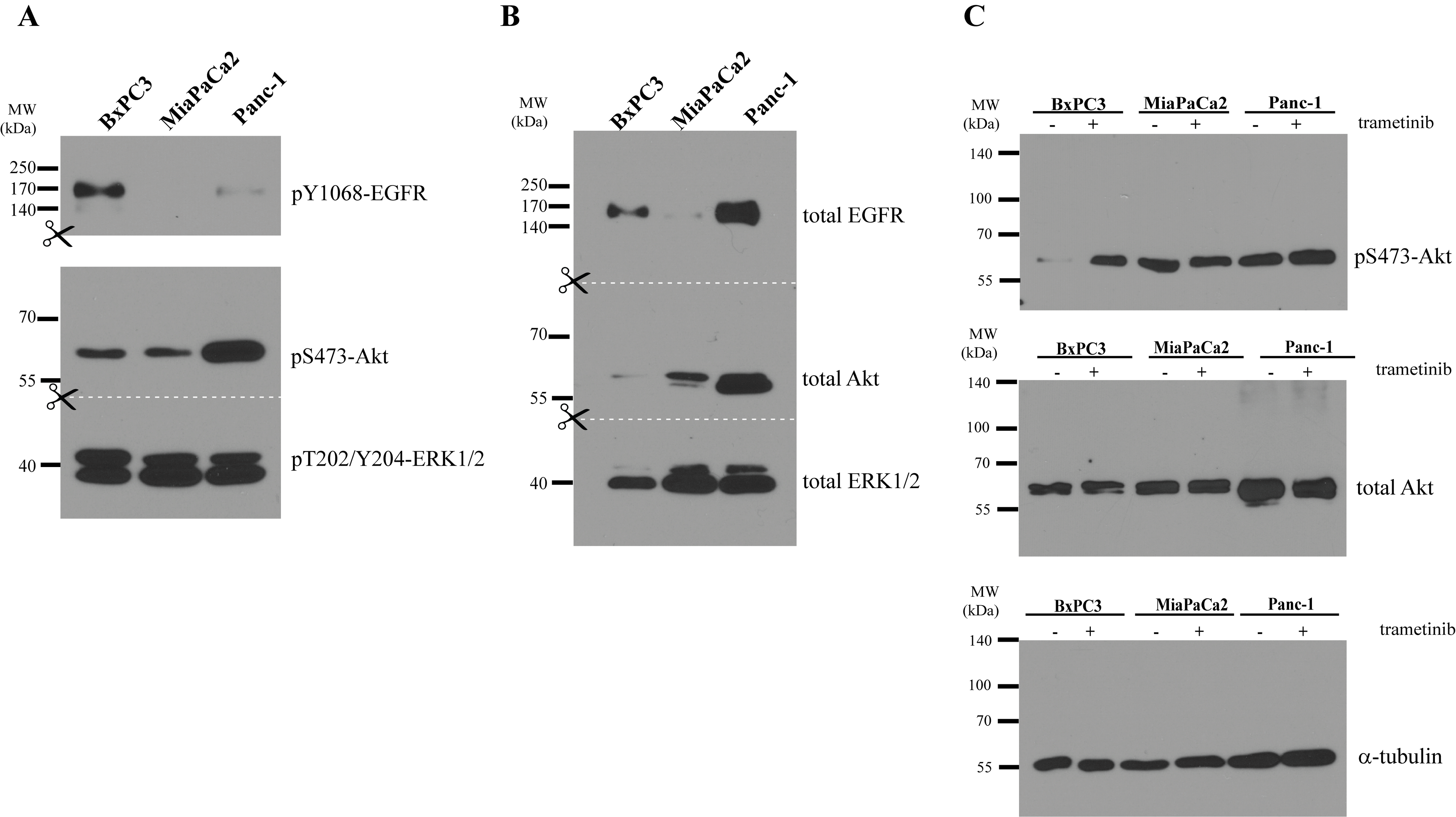

Supplement: S1 Fig — Figure A, B and C represent the original Western blot images used in Fig 3. and Fig 4. (TIF) [file pone.0185687.s001.tif]
